# Kinorhesis: A physiological principle of transformation that is complementary with homeostatic stability

**DOI:** 10.3389/fphys.2025.1633607

**Published:** 2025-10-10

**Authors:** Nelson D. Horseman

**Affiliations:** University of Cincinnati, Cincinnati, United States

**Keywords:** reproduction, development, negative feedback, positive feedback, quorum sensing, fruit ripening, metamorphosis

## Abstract

Since being articulated by Claude Bernard, and ultimately named by Walter Cannon, the theory of Homeostasis has been a conceptual and practical bedrock of physiology and medicine. Homeostasis addresses the idea that internal stability is a requirement for survival and proper functioning of organisms. A great variety of transformative changes associated with development and reproduction are not addressed by homeostasis. Two familiar examples of non-homeostatic transformative processes are metamorphosis and childbirth. In a previous paper the name “kinorhesis” was proposed for a physiological principle encompassing the processes that account for episodes of transformative changes during reproduction, growth, and development. Like changes brought about by natural selection, kinorhetic transformations can have profound effects on the fitness of the organism in new or changing environments. But unlike evolutionary changes, kinorhesis takes place within the lifetime of the individual rather than across generations. Kinorhetic transformative changes exist alongside homeostasis such that the physiology of kinorhesis articulates with that of homeostasis. In most instances homeostasis and kinorhesis exist cooperatively, but sometimes they come into conflict. This paper will elaborate the new theoretical framework of kinorhesis using examples from across the biological Kingdoms, and describing the types of physiological mechanisms that distinguish homeostasis and kinorhesis. Physiological regulatory processes that are responsible for homeostasis and kinorhesis include compartmentation, negative feedback (normalizing reflexes), positive feedback (amplifying reflexes), and sequential controls. Homeostasis and Kinorhesis set boundaries on processes that provide stability and transformation to physiology, and they provide an heuristic framework for discovery and education. Kinorhesis employs the full scope of physiology from cellular level functions through organ systems, behaviors, and morphological changes. All aspects of homeostasis and kinorhesis are consistent with conventional theories and processes of Darwinian evolution.

## 1 Introduction

Bernard (1885) was the first to articulate the importance of stabilizing physiological processes when he wrote that *“a free and independent existence is possible only because of the stability of the internal milieu”*. Several decades later Walter Cannon gave this principle the name “homeostasis” and provided detailed analyses taken from human and mammalian physiology (Cannon, 1929; 1939). The term homeostasis is derived from two Greek roots, “homeo-” meaning similar, and “-stasis” meaning stand or remain, so homeostasis addresses how physiological variables remain similar over time. Bernard and Cannon’s conceptions of homeostasis seem quite narrow today. Using the limited data of their times they conceived of homeostasis as applying only to warm-blooded animals (birds and mammals/humans), and only to the ingredients of the extracellular fluids (esp. blood plasma). Today homeostasis is observed in all the biological Kingdoms (3 types of microbes, 2 of plants, plus animals), and at all levels of organization from intracellular to tissues, organs, and organisms and collectives. Although Bernard and Cannon believed they had discovered physiological processes that made “higher” animals special, we now know that they had discovered a basic physiological principle of life (Horseman, 2025).

### 1.1 Kinorhesis: transformation via physiology

In a recent paper I introduced a new physiological concept that addresses how non-homeostatic transformative changes are caused and controlled (Horseman, 2025). This concept, named “Kinorhesis”, derives from Greek roots “kine-” meaning propel and “-rheo” meaning flow. Kinorhesis is defined as: “The physiological processes responsible for episodes of transformative changes that are necessary for reproduction, growth, and development.”

The current paper is intended to focus on biological details and examples to explore the nature of kinorhesis, and the ways in which it contrasts with and complements homeostasis. Because it is a new concept there will be many aspects of kinorhesis that are not yet answered.

The perpetuation of individuals, and their reproduction is conditioned upon physiological processes that result in transformative changes of function, morphology, and behavior. By definition, development and reproduction include transformative changes. Kinorhetic changes are often permanent, though some cycle back to a previous state. In some cases kinorhesis provides organisms the dynamic range needed to exploit multiple environments and styles of life that are optimal for different stages of development. Homeostasis and kinorhesis balance the risks and rewards of ceaseless stasis or unrestrained change.

The kinorhesis principle and homeostasis are complementary, not competing, concepts. Kinorhesis describes the processes that propel transformative changes whereas homeostatic adjustments maintain stability. The complementary nature of these two concepts means that kinorhesis does not contradict anything about homeostasis, but rather kinorhesis addresses aspects of physiology that are separate from those addressed by homeostasis. All of these complementary processes articulate to support the survival and perpetuation of organisms. Reproduction, growth, and development are inherently not static or stable. Under the general concepts of reproduction, growth, and development there are a limitless number of particular processes and mechanisms that have evolved, and will continue to evolve into the future. The principle of kinorhesis is strictly Darwinian (or neo-darwinian if you like). No mechanisms are required other than those that can be selected from the natural genetic variation that produces observable variations of physiology within a population.

The following section provides a summary of homeostasis and various controversies surrounding it. After this Section I will return to more discussion of kinorhesis.

### 1.2 Homeostasis: the stability portion of physiology

The theory of homeostasis has become a bedrock of not only physiology but also biology in general as it has widened and deepened in scope. Bernard’s original conception was that “stability of the internal milieu” was a special feature of warm-blooded macrofauna (birds and mammals/humans), and it provided these animals with abilities to be highly mobile and to engage in complex behaviors. Cannon and other physiologists before mid-20th century continued to be almost exclusively focused on mammalian systems. By the end of World War II concepts of control systems in animals and their analogues in engineering were formalized by Norbert Weiner and ultimately published as “Cybernetics: Or Control and Communication in the Animal and the Machine” (Weiner, 1961). Theoretical treatment of homeostasis and cybernetics made it possible to advance beyond describing examples of internal stability in mammals and begin looking for common control mechanisms across biology. As more was learned about the physiologies of invertebrates, plants, and microbes the theory and mechanisms of homeostasis became unifying ideas. The broad reach of the theory of homeostasis has been a relatively recent development, and controversies over the exact meanings of homeostasis have continued even as the theory has gained familiarity.

Homeostasis finds its greatest practical impact within the practice of medicine where restoration of homeostasis and preventing disruptions to homeostasis are primary imperatives. The values of physiological variables that are considered clinically homeostatic are referred to as “normal values”. These clinical normal values have a loose but informative relationship to the cybernetic term “set-point”, which physiologists use to designate a imaginary target value that the system will return to after having been disturbed.

Set-point and certain other terms (sensors, noise, controller, etc.) are shared between physiology and engineering and characterize physiological regulatory systems that exert control over aspects of the internal milieu. It is understandable that sharing terms between physiology and engineering is a challenge. The set-point of a device is known because the operator chooses it, whereas a set-point in physiology can only be inferred from the behavior of the system. Similarly, components like sensors, controllers, and effectors are concretely known in devices, but must be discovered and inferred in physiology or medicine.

The relationship between normal clinical values and the idea of homeostatic set-points is instructive. Normal values are expressed as a range rather than a point. Remembering that the “normal range” is a statistical description of a human population, not an individual, permits us to identify sources of the variability in this range. These include two sources within the individual: random variations through time (statistical noise), and variability caused by local environmental conditions (disturbances). Additionally there is variability among individuals in the population caused by heritable variations (Darwinian) and non-heritable variations (epigenetic).

When the values for a regulated variable move outside the normal range homeostasis has an increasingly difficult time and medical interventions can be needed to avoid decompensation (breakdown of homeostasis). Values for a physiological variable therefore may be in the normal homeostatic range (near the set-point), homeostatically perturbed (outside normal values but recoverable), or homeostatically decompensated (abnormal and requiring intervention). Romero et al. (2009) proposed a formal graphical model referred to as “reactive scope” in which variables could be within homeostasis, in homeostatic overload, or in homeostatic failure.

The success of homeostasis in the practice of medicine and the expansion of the theory to encompass all of the Kingdoms are important validations of the theory. Nonetheless, there have been many serious efforts to clarify what is meant by homeostasis and to offer alternative or replacement concepts. Fortunately, those efforts have been summarized and reviewed expertly in several major papers (Houk, 1988; Modell et al., 2015; McEwen and Wingfield, 2010; Turner, 2013; Billman, 2020; Bechtel and Bich, 2024; 2025 Davies, 2016). I do not intend to provide another analysis; instead I will draw from them to provide context for the ideas offered here.

One commonality among the papers that have explored alternative concepts and names for homeostasis is that their focus generally has been on issues drawn from mammalian organ system physiology. Early on Selye (1956) advocated using “heterostasis” when addressing mammalian physiological responses to stresses. The term “allostasis” was offered as an alternative for animals in not only stress but other arousal states (Sterling and Eyer, 1988). Davies (2016) suggested the term “adaptive homeostasis” for adjustments to set points during responses to stressors, toxins, or exertion. Dale Bauman used the term “homeorhesis” (Bauman and Currie, 1980) for physiological adaptations for pregnancy and lactation and implied that the concept could be applied to other circumstances. Using the term homeorhesis is somewhat problematic because the word was coined earlier by Waddington (1957) for a completely different idea. “Anticipatory homeostasis” or “predictive homeostasis” have been suggested as terminology for adjustments that accommodate predictable environmental changes such as daily (circadian) and seasonal or annual cycles (McEwen and Wingfield, 2010). In contrast, “reactive homeostasis” applies to responses to unpredictable perturbations. “Rheostasis” is a term proposed by Mrosovsky (1990). Rheostasis has similarities with allostasis but rheostasis is proposed to address normal changes in animal functions rather than pathophysiology. Rheostasis focused particularly on circadian and annual rhythms (Mrosovsky, 1990; Stevenson, 2023). Like allostasis and the other concepts outlined in these papers, rheostasis addresses how various changes in physiological variables and their regulation help to maintain stability over time and under differing environmental conditions.

Homeostasis has expanded its meaning to encompass the maintenance of stable functioning in all organisms and at multiple levels of organization. Thus bacterial and plant homeostasis are basic theoretical assumptions within microbiology and botany, respectively. Tissue homeostasis, meaning the regulation of cell numbers, cell sizes, shapes, and interactions is a core concept in cellular physiology and cell biology. Tissue homeostasis and the coordination of cell properties is an organizing principle for understanding wound repair, regeneration, neoplasias, and metastasis.

Although maintenance of stability is necessary for life, stability is not sufficient to explain all of physiology over the course of an individual’s life history. Various transformative events and processes are controlled by physiology and are necessary elements of a life history. Transformative processes in physiology are associated with growth, development, and reproduction. Evolution has provided organisms with homeostatic processes that provide stability for living in the now, and transformative (kinorhetic) processes that propel changes for growth, development, and reproduction so as to live and perpetuate into an uncertain future. Table 1 summarizes comparisons that explain the basic contrasts between the physiology involved in kinorhesis and homeostasis.

**TABLE 1:** Comparisons and contrasts between features of homeostasis and kinorhesis.

Homeostasis	Kinorhesis
Stabilizing	Transforming
Constant	Episodic
Not Morphogenic	Often Morphogenic
Quantitative	Qualitative
Universal	Universal

My approach in the remainder of this paper is the following: first I will describe, in some detail, an example drawn from nature that illustrates kinorhesis. This example is the migrations of salmon and it illustrates the interplay between homeostatic and kinorhetic processes through a life history. After that I will discuss basic physiological processes like feedback regulation that distinguish kinorhesis from homeostasis. I will return to additional examples of kinorhesis, drawing from not only animal physiology, but also from plant and microbial physiology. Finally, I will provide some perspectives on the heuristic impacts of the principles of homeostasis and kinorhesis on research and education.

## 2 Examplar: salmon migrating

The life histories of salmon exemplify and emphasize important aspects of the life cycle dynamism and transformations addressed by the kinorhesis framework. Salmon species in the Northern Pacific spawn in freshwater streams and spend their adult lives hunting and feeding in the ocean. This type of life history is called “anadromy”. Although there are differences among species, and even between local populations, anadromous salmon share a basic template of physiological and morphological changes through their life cycles. That life cycle template will allow us to illustrate kinorhesis and some of its relationships with homeostasis.

Nothing about homeostasis demands anadromy (life in both freshwater and seawater). Anadromy evolved multiple times as one of many life cycle strategies to deal with competition among fishes. In the case of salmon the adults are able to take advantage of vast populations of prey fish in the oceans (Dodson et al., 2009). A great many fish species spend their entire lives in either freshwater or seawater, unable to adapt to the alternative but perfectly capable of homeostasis in their natural environments. When fish are homeostatically adapted to only freshwater or seawater they are referred to as “stenohaline” (steno-: narrow, -haline: salt). Anadromy, in its contrasts with stenohaline homeostasis, illustrates transformations during a life cycle with a familiar vertebrate example.

**FIGURE 1:**
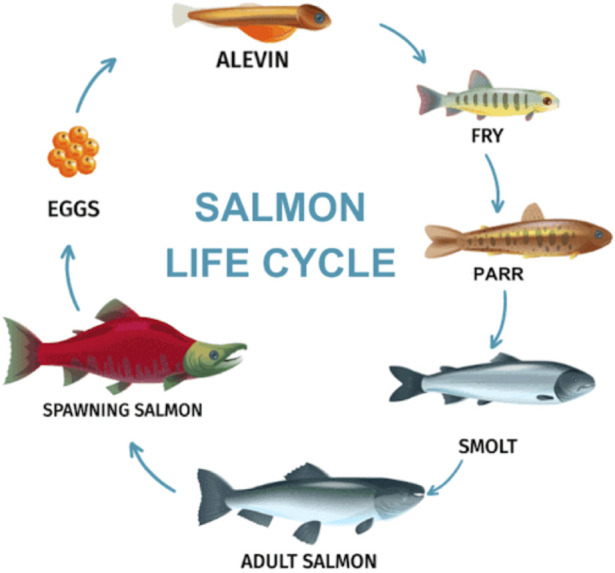
Generalized life cycle of Pacific Salmon (https://natureroamer.com). See text for descriptions.

To complete their life cycles juvenile salmon migrate downstream to the sea, and then after they have grown and fattened the adults migrate back up their home streams to spawn (Figure 1). These migrations require two different transformative programs, with many imbedded subroutines. During downstream migration the salmon undergo major physiological changes that are necessary for new environments. During their upstream migration the salmon abandon many aspects of homeostasis and die before, during, or after spawning. Kinorhesis encompasses these processes. Reproductive and developmental transformations that take place during salmon migrations employ the full scope of physiology from cellular level functions through organ systems, behaviors, and morphological changes.

After salmon eggs hatch they go through three juvenile stages in which they are stenohaline freshwater residents. The first stage, alewives, carry the yolksac from their eggs for nutrition. Alewives transform into fry, which are free living and feed on tiny plankton. The fry are weak swimmers and stay among pebbles in the bottom of their stream where there is little or no current. The final juvenile stage are called parr, which are also strictly freshwater. Parr feed on larger prey and are camouflaged with bronze colors and vertical bar markings suited for their streams. The osmoregulatory systems of these juvenile stages are typical of freshwater fishes. They absorb a lot of water passively, which is excreted as large volumes of very dilute urine. Salts are absorbed from their food and efficiently conserved by the kidneys and the gills. Active transport (ion pumps) and passive solute movement through selective channels in the kidneys and gills have been expertly reviewed (Edwards and Marshall, 2012; McCormick, 2012). The gills and body surfaces are generously coated in mucous that moderates water and solute movement into or out of the animal.

Parr transform into a form named “smolt”. This transformation is very critical for the salmon industry, so it has been studied more than the juvenile transformations (Hoar, 2024). The salmon swim and float downstream while their physiology is being transformed to that of the adult. Some species may travel 3,000 km from where they were hatched before reaching the ocean. The transformation from parr to smolt is gradual and therefore is usually referred to as “smoltification”. During smoltification overall body shape is remodeled and there are major functional and morphological changes to a variety of organs; especially the gut, integument, gills, and kidneys. An obvious change is the replacement of the drab stream camouflage coloration of the parr with reflective coloration, known as silvering. The silvery appearance is caused by deposition of hypoxanthine and guanine crystals in the skin cells and scales. This coloration will be suitable to the adults for open water and swimming in schools. Along with coloration, the body is remolded into its efficient streamlined torpedo adult shape.

A remarkable physiological change during smoltification is their transformation from the stenohaline freshwater juveniles into euryhaline adults (eury-:broad, -haline: salt). Osmoregulation in seawater requires the fish to drink lots of saltwater and excrete salt efficiently, and to conserve body water against an external environment with an osmotic pressure more than 3-times higher than the internal. One of the more interesting transformations is of the esophagus, which becomes able to absorb large amounts of salt from seawater the salmon drink copiously. The salt is circulated to the gills where it is excreted by specialized ionocytes. These ionocytes (also called chloride cells) are densely packed with mitochondria that provide energy in the form of ATP to power molecular pumps to excrete salt. The smolts will remain in the brackish estuaries for some period of time while the biochemical and morphological changes necessary for oceanic life are completed. Two endocrine hormones that have important roles in smoltification are cortisol and growth hormone (Boeuf et al., 1994; Takei and McCormick, 2012). Other hormones are surely involved but insufficient research limits what is known.

As the smolt transformation proceeds during downstream migration the fish imprint on chemicals in their natal streams, and these signals along with others will guide their return to spawn. The primary imprinting chemicals are amino acids, the composition of which can vary substantially from stream to stream. Other bio-organic chemicals (bile acids, fatty acids, etc.) play subsidiary roles (McCormick, 2012).

Adults will range widely in the oceans feeding, growing, and fattening for several months to a few years before reaching a size and reproductive maturity that propels them to undertake their return migration. Gonadal development is driven by gonadotropin-releasing hormone (GnRH) from the hypothalamus, and gonadotropic hormone from the pituitary gland. The steroid hormones from the maturing gonads, especially testosterone, stimulate transformations in physiology, morphology, and behavior.

The spawning migrations begin with the adults navigating to the estuaries at the mouths of their home rivers, using primarily magnetic and visual cues (Putman et al., 2013; Quinn et al., 1989). They use the chemical signature that was imprinted during their downstream migration to navigate once they are near the drainage of their home streams.

When the salmon arrive at the entrance to their home stream they stop feeding. Having spent months or years voraciously hunting and feeding they now switch off their appetites. Practically nothing is known about how this remarkable change comes about for a basic homeostatic system. They will use stored nutrients for a journey that can be as long as 3,000 km upstream and as much 2,000 m in elevation. To succeed their stored nutrients will also need to suffice for maturing the gonads, competing for spawning sites, and finally spawning. During the migration most calories come from fat stores (75%+) whereas anaerobic glycolysis provides short bursts of energy when negotiating rapid currents and waterfalls. Proteolysis is induced, presumably by cortisol, and the amino acids support tissue remodeling, repair and gluconeogenesis (Miller et al., 2009; Mommsen, 2004).

**FIGURE 2:**
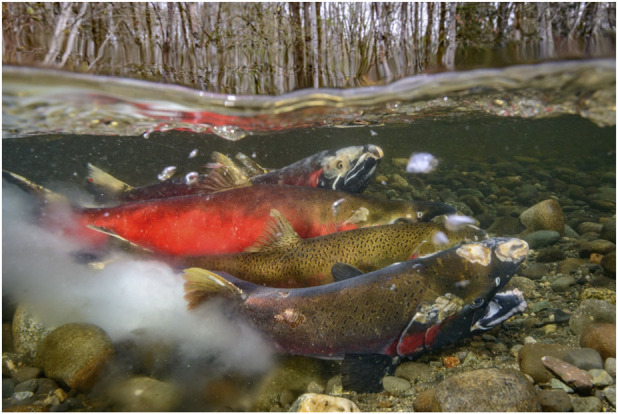
Spawning salmon. Female attended by two males as she deposits eggs in a redd and the males fertilize with milt. Notice the many unhealed injuries and sores. Photo by Eiko Jones, used with permission.

While the salmon migrate upstream there are numerous transformations to their physiology and morphology. Gonadal steroids drive changes in body shape and coloration that are somewhat species-specific. Males undergo the most visible changes. The head and jaws of males undergo major changes resulting in the formation of the “kype”, a hooked, elongated, and toothy elaboration of the snout that is used in territorial fights and displays (Figure 2). The kype varies in size and shape among different species. The female head changes less dramatically, but the changes are important for nest building and defense. The salmon loose their silvery coloration and change to bright colors that are part of the reproductive display system for attracting spawning mates. Some changes are species-specific, such as the large dorsal hump that grows just behind the head of male pink salmon.

The immune system of salmon is suppressed during the spawning migration. High cortisol levels throughout the spawning migration are believed to be immunosuppressive, but there are certainly other hormone and cytokine systems involved. Antibody levels are somewhat lower and innate antibacterial proteins decline markedly (Dolan et al., 2016). Little is known about changes in cellular immunity.

At the spawning beds the salmon compete strenuously and violently for real estate and mates. The females scoop out a depression called a “redd” and lay up to several hundred eggs, which the males fertilize (Figure 2). Depending on the species each female may produce multiple redds and deposit up to several thousand eggs in total.

**FIGURE 3:**
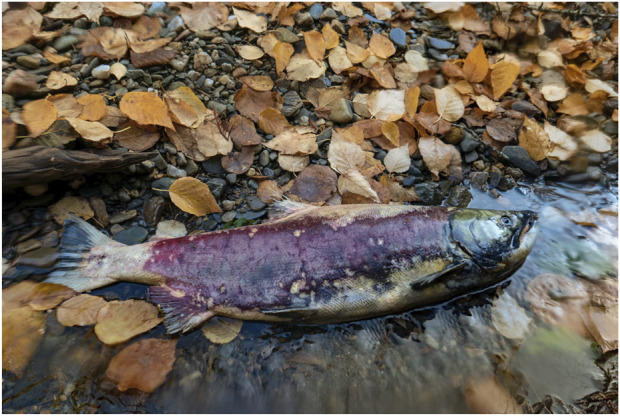
Female Sockeye salmon that has recently died on the spawning grounds. Photo by Sharif Galal, used with permission.

The linear narrative of the salmon migration typically says that after spawning the adults die. And it is true that all the adults that survive to spawn will die soon thereafter. But a more complete narrative is that those adults that begin the spawning migration have a high risk of dying during the migration because they are very poorly suited to living in these streams. To begin with, the adults are simply too large to live in the streams where they will eventually spawn. Some of the adults will not have sufficient energy stores to complete the migration, others will die from injuries sustained at the rapids, dams, and waterfalls they must negotiate. Some will die before spawning because their suppressed immune system fails to fight off infection. Many will fall prey to bears, wolves, coyotes, birds, and humans, and their lack of camouflage contributes to this risk. And much of their homeostasis will be abandoned during the migration in favor of a whole suite of irreversible physiological, morphological, behavioral, and developmental commitments to reproduction. The adults that reach the spawning grounds will pour their remaining resources into reproducing, then they too will die (Figure 3).

The migrations of salmon clearly illustrate that homeostasis alone cannot account for the transformative physiological processes that constitute a complete and complex life history. There are drives that propel major functional transformations, including but not limited to, changes in homeostatic regulation (e.g., stenohaline-to-euryhaline). These transformative physiological processes live alongside and sometimes come into conflict with homeostasis.

We turn now to the types of regulatory mechanisms underlying physiology, and how differences in those mechanisms can help to clarify the boundaries between homeostasis and kinorhesis.

## 3 Homeostatic mechanisms

Life is possible because organisms maintain internal states within survivable limits. The homeostasis principle encompasses a seemingly limitless variety of short to intermediate term reactions that maintain internal conditions within acceptable tolerances. Homeostasis accomplishes two essential tasks. First, to create internal conditions that are compatible with life in the face of external environments that are hostile. These include a highly oxidizing atmosphere and surroundings that are either too dry if you are a terrestrial being or too wet if you are aquatic. Secondly, homeostatic processes resist large or rapid changes to the internal milieu.

The “internal milieu” that Bernard and Cannon were focused on was the fluid that surrounds the cells of the body (extracellular fluid, ECF), and Cannon was narrowly thinking about the physiology of humans and other mammalian animals that he studied (Cannon, 1939). Today biology is able to take a comprehensive view of internal vs. external environments from the subcellular to organismal levels and across the living Kingdoms.

Despite the number and variety of individual physiological processes that have been discovered there are two types of mechanisms that provide the basis of homeostasis. The first is compartmentation within functioning boundary membranes, which define the external and internal environments at the root of homeostasis. These boundaries are commonly nested so that internal vs. external zones are organized hierarchically. This provides for the most internally situated zones (intracellular and organellar) to be the most stably regulated. The second category of process is regulatory negative feedback loops, which couple physiological variables into regulatory relationships that maintain homeostatic limits. Homeostasis would not be possible without compartmentation and negative feedback although additional physiological processes are involved in specific situations.

Biological membranes operate both passively and actively, and they can be very small (plasma membrane of a *mycoplasma*) or very large (skin of a whale). Regardless of their size and structural complexity biological membranes all contain a mix of passive and active elements. Here we will be concerned with illustrative features of biological membranes. More complete information about cellular, tissue, and integumentary membranes are found in many excellent physiology textbooks, including Boron and Boulpaep, 2016, Koeppen and Stanton, 2023, and Hall, 2021.

The fundamental membrane is the plasma membrane that surrounds individual prokaryote and eukaryote cells. Its familiar phospholipid bilayer structure is impermeable to water and dissolved ionic solutes but for the fact that the membrane is penetrated by a variety of proteins that provide passive and active pathways for the internal milieu to exchange particular products with the external. This selective and regulated exchange with the outside is the basis of cellular homeostasis. Passive features of plasma membranes are their impermeabilities to water, ionic solutes, and most large molecules. Active features in plasma membranes are absorption and secretion of substances for metabolism, excreting wastes, and communicating with other cells and tissues.

Plasma membrane proteins that serve as selective diffusion routes for important molecules represent one class of homeostatically important membrane proteins. Glucose transporters (abbrev. GLUT) are an example. The GLUTs act as channel proteins selectively facilitating the diffusion of glucose down a concentration gradient from outside to inside. GLUT proteins provide a rudimentary type of metabolic integration by linking glucose uptake with the enzymatic conversion of glucose to other molecules, which drives intracellular glucose levels low enough to create a large concentration gradient from outside to inside.

Active transporters, such as Na^+^-ATPase are membrane proteins that cleave high energy phosphate bonds of ATP and undergo conformational changes that move solutes up a concentration gradient. Multiple Na^+^ transporters and cotransporters cause intracellular Na^+^ levels to be very low, and create a large gradient of extracellular to intracellular Na^+^. This electrochemical gradient is one of the primary determinants of cell excitability and provides motive force for many transport processes.

The plasma membrane interacts with cytoskeletal proteins in the determination of cell shape and motility, and formation of cilia and flagella. Proteins in the plasma membrane include receptors that detect hormones and neurotransmitters, and contribute information that distinguishes self from non-self.

The relative stability of both the intracellular and extracellular fluid spaces are, in large part, consequences of the collective functions of plasma membranes.

Boundary membranes occur at all levels of physiological organization, not only cell membranes. There also are tissue membranes that segregate cell types such as epithelial from adipose and that separate tissues from the interior of blood vessels and other ducts. Organs such as muscles and intestines are surrounded by strong connective tissue membranes. And whole organisms are held within membranes, cell walls, exoskeletons, and skins. Segregation of an internal milieu that is homeostatic is the physiological consequence of these biological membranes.

**FIGURE 4:**
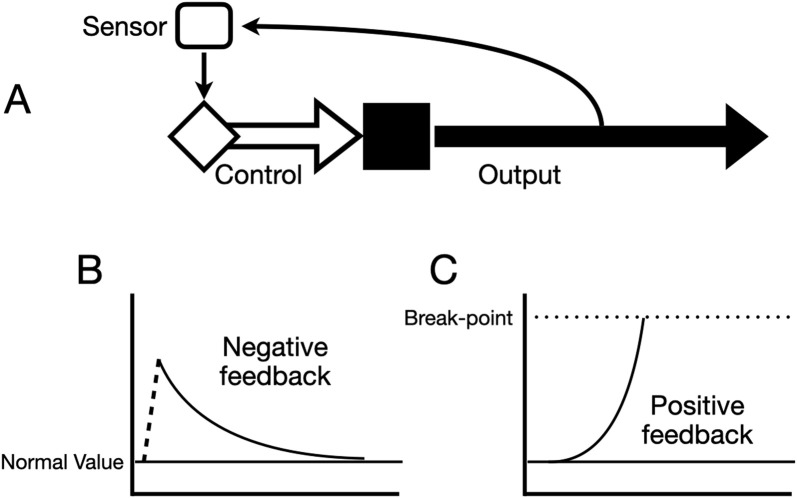
Diagrams of basic schemes for regulatory feedback loops. **(A)** The system output (secretion of a hormone, absorption of a nutrient, etc.) is detected by a sensor that may be a particular cell or group of cells or an interacting ensemble. The sensor controls the output of a regulatory signal (neural signals, hormones, etc.) that exerts either negative or positive control over the system’s output. **(B)** Idealized response of a negative feedback loop. A disturbance (dotted line) changes the value of the output and the feedback causes the output to gradually return to normal (set-point). **(C)** Idealized response of a positive feedback loop. The output moves away from normal increasingly until it reaches some end state (break point).

Negative feedbacks are the second category of physiological mechanisms that underlie homeostasis. Negative feedbacks maintain stability by coupling a regulated variable (extracellular osmolality for instance) to a reflex loop that includes the means of its regulation (e.g., hormones and kidneys) (Figures 4A,B). These regulatory loops are required for an organism’s defense against changes that could push it beyond sustainable limits.

**FIGURE 5:**
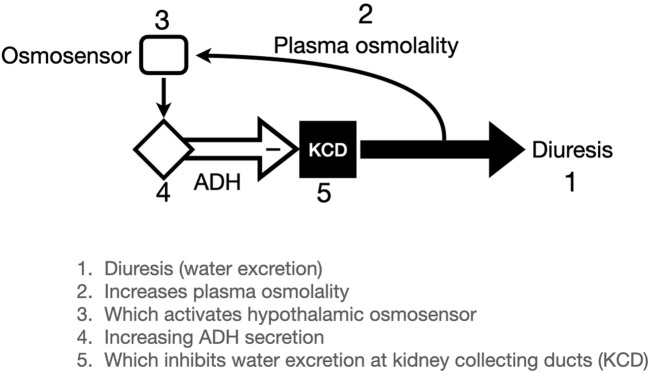
Illustration of Antidiuretic Hormone (ADH) negative feedback loop. Plasma osmolality is determined (in part) by water excretion (diuresis). Osmolality of the extracellular fluid affects osmosensitive cells in the hypothalamus which are linked to (probably identical to) ADH secreting neurons. When osmolality increases, ADH secretion inhibits diuresis by favoring water conservation in the kidneys.

An example of the operation of a negative feedback in mammalian physiology is the regulation of extracellular fluid osmolality by the brain hormone ADH (antidiuretic hormone) and the kidneys (Figure 5). ADH is a small peptide hormone (9 amino acids) that is synthesized in neuroendocrine cell bodies in the hypothalamus and transported down their axons to the posterior lobe of the pituitary gland. The ADH neurons are osmosensitive such that increases in extracellular fluid osmolality cause increased ADH secretion, and decreases in osmolality reduce ADH secretion. ADH opens water channels in the kidneys so that water is absorbed from the tubular fluid to decrease ECF osmolality. Reciprocally, low ECF osmolality reduces ADH secretion and water reabsorption in the kidneys, resulting in diuresis, dilute urine and recovery of ECF osmolality (Carmody et al., 2015).

The conventional analogy to illustrate negative feedback regulation is a thermostat in your home, which turns on the furnace when the temperature falls, and shuts off the furnace when the temperature returns to the set-point. This is called, unsurprisingly, “on-off” control. The ADH reflex described in the previous paragraph is as close to this thermostat analogy as any homeostatic biological reflex that we know. Below about 280 mOs/kg ADH secretion is undetectable, and when plasma osmolality increases by as little as 1% there is detectable ADH secretion and the slope of ADH increase is very steep. So, this resembles an on-off negative feedback with a set-point of about 280. Of course, nothing in biology is ever that simple, and ADH is controlled by a separate feedback system involving blood pressure (hence the alternative name, vasopressin). This “baroreceptor” reflex has different neural pathways and different reaction kinetics. In addition, both blood pressure and ECF osmolality have additional regulators other than ADH/vasopressin. Keeping in mind that these systems evolved through innumerable environmental challenges, we ought not be surprised at complexity that is very different from any human engineered regulation system.

Biological negative feedback loops generally operate along the lines of “proportional controllers” rather than simple on-off controllers. These types of feedback controllers are used extensively to engineer self-regulating devices (motors, etc.). A familiar example is an automobile cruise control system. Proportional controllers are designed to send a regulatory signal (gain) that is proportional to the deviation from the set point (error). Therefore, as the car gets closer to the set point the regulatory gain decreases proportionally and achieves a smooth change of speed. Control of blood glucose approximates a proportional control system wherein the level of insulin secreted is proportional to blood glucose. Again, nothing in biology is ever simple and there are at least 3 other hormones involved in glucose control and insulin itself is secreted in both a rapid phase and a slow phase. But if an analogy that is approximate helps us understand the system, that’s all to the good.

The stabilizing effect of homeostasis is universally appreciated in biology, and homeostasis is a main theme in the teaching of physiology. But homeostasis does not account for all of the physiological processes that are required to maintain and perpetuate lives. Reproduction, growth, and development are not caused by homeostasis. Even though homeostatic processes operate during reproduction, growth, and development, homeostasis does not cause these changes. These non-homeostatic physiological processes require a different theoretical framework, for which the term kinorhesis is proposed.

## 4 Kinorhetic mechanisms

Kinorhesis propels organisms in various ways depending on how evolution has shaped their life history strategies. As illustrated by the salmon life cycle, growth, development, and reproduction are physiological processes that are kinorhetic rather than homeostatic. Metamorphosis is a developmental consequence of physiological kinorhesis. Kinorhesis, like homeostasis, provides a framework for categorizing physiological processes based on biological consequences and causes.

An obvious distinction between homeostatic and kinorhetic regulation is that homeostatic processes operate continuously in their particular contexts. Osmolality, nutrient levels, hormone concentrations, body temperature, etc., are monitored and regulated continuously. Kinorhesis, by contrast, is inherently episodic. Intervals of obvious transformative changes interrupt spans of relatively quiescent stability. The episodic nature of kinorhesis is a consequence of the control processes that are responsible for kinorhetic transformations.

Kinorhetic regulation is characterized by two well understood types of control processes, Positive Feedback Loops and Sequential Control. Like negative feedback these regulatory processes have physical analogues that are used routinely for engineering applications. Regulatory actions other than positive feedback and sequential control take place during kinorhetic processes, but these two types of regulation seem to be diagnostic for kinorhesis.

As the name implies, positive feedback is the opposite equivalent of negative feedback. A change in the regulated variable causes additional change in the same direction, reinforcing and amplifying the variable (Figure 4C). Organisms amplify small initial changes into much larger outcomes by employing positive feedback. These amplified outcomes are important to force non-homeostatic changes for organisms. If unchecked, positive feedback can result in a catastrophic runaway, as illustrated by chemical or nuclear explosions. Fortunately in physiology positive feedbacks are generally limited by the system reaching an end-state.

Childbirth is a familiar human physiology example that uses positive feedback. The end state for amplifying uterine contractions during childbirth is delivery of the baby and placenta. A familiar positive feedback example in plants is signaling by ethylene, which stimulates fruit ripening (Yamamoto et al., 1998). The positive feedback makes it more likely that fruits will ripen within a narrow window of time. Movement of microorganisms upwards along a concentration gradient (chemotaxis) uses positive feedback (Wadhams and Armitage, 2004). Electrochemical excitation of cells employs a positive feedback system that was mathematically described by Hodgkin and Huxley (1952). Muscle contraction relies on calcium-induced calcium release (i.e., a positive feedback) to cause rapid twitching of the myocyte. Though each of these is an example of positive feedback, some of them, like a myocyte twitch are not transformative and therefore would not be considered kinorhetic.

Positive feedback as a kinorhetic mechanism for propelling biological events finds particularly impactful expression in colonial insects, such as honeybees. Navigating to a food source or to a swarm destination is propelled by chemical and behavioral cues that are transmitted to members of the colony in chain reactions that can encompass the entire colony (von Frisch, 1927; Seeley and Visscher, 2004). These highly coordinated and vectorial behaviors are initiated from random searches by individual members of the colony and become highly non-random by virtue of positive feedback and competition among signals from multiple explorers.

As the examples illustrate, positive feedback is a form of regulation that has evolved at all levels of biological organization from subcellular to colonial.

Sequential control mechanisms are central for causing life histories to play out in highly ordered and predictable patterns of growth, development, and physiological adaptation. Another term for this type of regulation would be feed-forward, which would have the benefit of a parallel engineering term. The phrase feed-forward (or feedforward) has been used in different ways within biology (Houk, 1988; Modell et al., 2015). Sometimes feed forward has been used as synonymous with positive feedback, and in other cases it has been used as an alternative term for “preadaptation”. Consequently, the terminology used here for clarity is “sequential control.”

**FIGURE 6:**
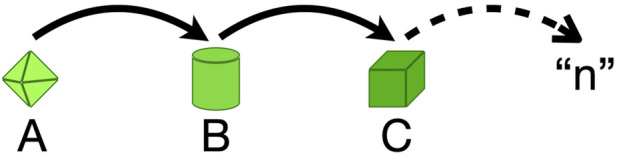
Conceptual schematic of sequential control. A sequence of physiological (morphological, behavioral, metabolic) states that are different in kind are represented by items **(A–C)** and an indefinite number (n). Subsequent states are initiated by some signal(s), such as achieving a critical size, or a critical level of a chemical signal.

Sequential control occurs when an output initiates a qualitatively new state or series of states (Figure 6). Sequential control results in an organism or tissue or cell that is different in kind from its original state, and usually includes a suite of multiple changes. Positive feedbacks and sequential controls often work hand-in-hand with positive feedback destabilizing an existing homeostatic state and sequential controls imposing one or more new states on the system.

Sequential control logically resides outside the bounds of homeostatic processes. Homeostatic processes are defined as those that entail the maintenance of stability or returning to an optimal state after some excursion from that set-point. In contrast, sequential control processes propel the system from a pre-existing state to a new state with at least some new homeostatic requirements. This logical distinction does not, of course, mean that homeostasis and sequential control cannot exist side-by-side at any given time in an organism’s life history. In fact, accomplishing a change through sequential control without surrounding homeostatic mechanisms would obviously be dangerous. The examples of the two salmon migrations illustrate this point. In the one (downstream), sequential control mechanisms revise homeostasis so the smolts successfully adapt to new environments. In the upstream migration the mature adults abandon many aspects of homeostasis while going through migration and reproduction, which leads ultimately to disease and death.

The term “anticipatory homeostasis” has been proposed to for changes that prepare the organism to cope with future conditions (Modell et al., 2015). Converting osmoregulation in salmon smolts from stenohaline to euryhaline could potentially be an example of anticipatory homeostasis. While this terminology may not seem particularly objectionable it is definitely problematic. Anticipatory homeostasis is a teleological phrase that implies that the cause of the adaptation is the “anticipation” rather than a physiological process encoded by sequential control regulation. Evolution has genetically programmed physiological events such as hormonal induction of osmoregulatory proteins. But the animal does not “anticipate” that those proteins will be needed. The organism is physiologically playing out its life cycle program. Sequential control terminology places causality properly before the developmental or acclimatization events.

Sequential control mechanisms that are responsible for anterior-posterior differentiation in insects are imprinted directly in the genome. The series of 8 homeobox genes that control the segmental insect body plan are linearly arrayed on a single chromosome. These Hox genes are spatio-temporally activated to direct the proper development of the body plan. In mammals 4 Hox gene clusters comprise a set of 39 genes that guide the anterior-posterior developmental plan (Gehring and Hiromi, 1986; Carroll, 1995; Hajirnis and Mishra, 2021).

Development of the organism is built upon sequential control mechanisms. These mechanisms can misfunction because of genetic mutations, toxins, or nutritional causes. Thyroid hormone synthesis requires dietary iodine. Thyroid hormone is a developmental signal that is required for a suite of changes that must occur in late fetal or early postnatal development (Medeiros-Neto and Rubio, 2015). Deficiency of dietary iodine during pregnancy interferes with normal development, resulting in severe mental and physical defects.

The key takeaways are that homeostasis and kinorhesis can be defined and characterized on the basis of their association with different physiological outcomes and different physiological mechanisms. Homeostatic outcomes are stability and relative constancy whereas kinorhetic outcomes are functional, morphological, and behavioral transformations. Homeostasis relies on compartmentation (creation of an internal milieu) and negative feedbacks (stabilization of internal milieus). Kinorhetic processes are positive feedbacks (destabilization of existing states) and sequential control (creation of new states). These physiological principles are useful because they can be defined with clarity and specificity. With these theoretical ideas in mind we can look a few specific examples of kinorhesis in different groups of organisms.

## 5 Biological illustrations of kinorhesis

In this Section I want to illustrate kinorhesis with examples from animals, plants, and microbes. If kinorhesis, like homeostasis, is a fundamental governing principle in biology we expect to find examples across the spectrum of species. The challenges involved in studying physiology are such that the particular processes are known in only a limited number of species, some of which are summarized here.

### 5.1 Metamorphosis

Through metamorphosis organisms are propelled from one state with its own homeostatic rules and mechanisms into a new state in which homeostasis is profoundly changed (tadpoles live in water and toads live in air). As a life cycle strategy metamorphosis is very common among invertebrates, and a defining feature of Amphibian vertebrates. Conventionally we think of metamorphosis in terms of the associated morphological changes, which can easily be documented in living or dead specimens. But morphology is only one expression of the changes created and controlled by physiological processes.

The most studied and best understood metamorphic animals are insects. Those that go through complete metamorphosis have 4 life stages: egg, larva, pupa, and adult. Larval insects engage in feeding and growing and adults engage in finding mates and reproducing. During the pupal stage the larval organs are dissolved and adult organs are formed. An obvious but important feature is that the stages are deterministically vectorial. The insects cannot go directly from larva to adult, nor can they go back to being pupae after emerging as adults. Larvae must store sufficient nutrients to survive at least the pupal stage. And in many species the adults go through mating and reproducing without feeding.

Even after decades of highly successful studies the details of metamorphosis in both insects and amphibians are incompletely understood at the cell and tissue levels. What is clear is that metamorphosis involves complex physiological signaling interactions that are very different from the well-understood development of the nematode, *Caenorhabditis elegans*. Whereas *C. elegans* development is linear and very much “hard-wired” (Sulston, 2003), metamorphic development is plastic and tolerant of interruptions and interventions (Vandenberg et al., 2012). Although not strictly metamorphic, the regulation of development in humans and other mammals is likely to be much more similar to amphibians than to nematodes.

Metamorphosis is based on innumerable specific regulatory events and interactions that accomplish a series of control processes. Pupation initiates when the final larval stage (last instar) reaches a permissive body mass (called “critical weight”) (Yamanaka et al., 2013). Growth of the last instar larvae is a function of food availability and hormonal control, primarily by the insect insulin/IGF system. Once the critical weight has been realized, the juvenile hormone (JH) level declines and prothoracicotropic hormone (PTTH) increases, which stimulates ecdysone synthesis and secretion from the prothoracic glands (Mirth et al., 2005). Ecdysone induces expression of its own receptors in many tissues, amplifying and accelerating the signal. The ecdysone signal is extinguished by two mechanisms that are induced by ecdysone in target cells, export and metabolic breakdown of ecdysone (Yamanaka et al., 2013). The combination of amplifying the signal and inducing the extinguishing of the signal results in a pulse of stimulus that synchronously times metamorphosis.

### 5.2 Vascular plants

Plants may not undergo transformative events that appear to be as dramatic as migration or metamorphosis, but the principles of homeostasis and kinorhesis apply equally to the plant life cycles. Economic considerations have promoted research in certain species and particular aspects of plant physiology. Consequently, a well understood example of kinorhetic regulation in plants is the physiology of fruit ripening (Yamamoto et al., 1998). Fruit ripening is a phase during plant reproduction when a variety of tightly regulated non-homeostatic changes occur to physiology, morphology, and biochemistry in the extracellular and intracellular environments of the plants.

Plants fall into two categories, climacteric, which ripen rapidly and synchronously (e.g., bananas) and non-climacteric, which ripen gradually and asynchronously (e.g., strawberries). The basic scheme that controls ripening involves the hormones auxin (indoleacetic acid), which inhibits ripening, and ethylene, which stimulates ripening. Ripening is a sequential control process that involves the expressions and activities of biosynthetic enzymes, receptors, signaling mediators, and effector molecules. Intermediaries in the process include homeodomain proteins, related to developmental homeodomain proteins of animals. Similar to animals, these transcription factors mediate sequential gene inductions that determine the order of events in development.

Ethylene secretion is mediated by a basal system (system-1) which is controlled by ethylene through negative feedback, and an induced system (system-2) which is controlled by positive feedback of ethylene. System-1 is responsible for ethylene synthesis during normal vegetative growth and ripening in non-climacteric fruits. System-2 is employed by climacteric fruiters. It is autoinduced (positive feedback) by ethylene via activation of transcription factors that drive the expression of specific isoforms of ethylene biosynthetic enzymes (Lin et al., 2009).

Abscission (Latin: cut off) is a kinorhetic process by which plants shed parts such as leaves and fruit. Like fruit ripening, abscission is an aspect of plant life histories that is non-homeostatic in the sense that elements of the internal environment are not stable, and the plant undergoes changes from its current homeostatic circumstances to a new set of states, which can be the death of parts of the plant such as leaves or flower petals.

### 5.3 Bacteria

Quorum sensing is a name for a variety of bacterial cell-to-cell communication processes that enable the bacteria to alter their behaviors (physiology, morphology, motility, virulence, etc.) in response to environmental conditions. Biofilm formation and patchiness, virulence, and bioluminescence are outputs controlled by quorum sensing. The signaling factors that drive quorum sensing are termed “autoinducers” because they create positive feedback loops by inducing their own biosynthesis. Autoinducers are small diffusible molecules. In Gram-positive bacteria most autoinducers are acylated, enabling them to cross the cell membranes and bind to transcription factors in the bacterial cytoplasm, whereas Gram-negative autoinducers are often small peptides that interact with membrane associated receptors (Suntharalingam and Cvitkovitch, 2005; Papenfort and Bassler, 2016). Positive feedback assures that a community of bacteria can accomplish changes that individuals could not, and that an integrated bacterial community is local with multiple potentially heterogeneous communities over a large area such as a mature biofilm.

One way to contextualize the kinorhetic nature of quorum sensing is by reference to the concept of internal milieu. In a planktonic population of bacteria each individual has a homeostatically-regulated intracellular compartment that interfaces via the cell membrane with the external environment. Quorum sensing creates a community, such as a biofilm, that now has a colonial “milieu” that is modified and maintained locally by the collective actions of the population. The fact of this rudimentary interstitial space can be inferred from responses to locally secreted autoinducers, and to effects of fluid flows and fluid stasis on the colonies (Suntharalingam and Cvitkovitch, 2005). Quorum sensing therefore propels the bacterium from a free-living state with a single internal milieu to a communal state with multiple layers of internal milieu.

Quorum sensing systems are organized in control cascades in which not only does an autoinducer drive its own synthesis, but it also drives downstream autoinducers that have receptor-transcription factor partners that activate additional sets of effector genes (Papenfort and Bassler, 2016).

While the language of quorum sensing is generally applied to bacterial cell-to-cell communication, the conceptual principles are not very different from systems in multicellular eukaryotes. Complex systems as diverse as autocrine-paracrine signaling in tissues and behavioral or pheromone signaling among colonial insects can be modeled on the same concepts as bacterial quorum sensing (Papenfort and Bassler, 2016; Chen et al., 2015).

Sporulation, or more precisely endospore formation, is another example of kinorhetic physiology in bacteria. Unlike spores of algae and plants, bacterial endospores are not reproductive. Bacterial sporulation is purely a dormancy mechanism that occurs under stress. The process has been most extensively studied in *Bacillus subtilis*, a gram positive rod-shaped bacterium inhabiting soil and the enteric tracts of various species (including cattle and humans). When threatened by adverse environmental conditions (high temperature, desiccation, crowding) *B. subtilis* undergoes sporulation, forming a very tough dormant form of the cell that can withstand extreme environmental conditions and remain viable for long periods of time. Endospore formation is a type of bacterial differentiation that begins with asymmetric cell division and leads to encasement of the daughter cell within the resilient endospore membrane/wall. The dynamics of the process stem from autoinduction of a master regulator (Spo0A) which additionally drives autoactivation through a complex phosphorelay system that includes a second regulator, Spo0F (Mitrophanov and Groisman, 2008). This system of positive feedback and sequential control propels the rapid kinetics of endospore formation, and creates locality in the endospore response so that only a fraction (generally less than 10%) of the population forms spores (Strauch et al., 1993). The heterogeneity of sporulation allows the population to exploit multiple survival strategies simultaneously.

Tocheva et al. (2016) proposed, on the basis of molecular and morphological details, that early during evolution a cataclysmic event, or multiple events, caused a survival bottleneck after which the only survivors were bacterial spores. The progeny from the surviving spore(s) were the common ancestors of all of subsequent life. Lineages that might have been well adapted to the preexisting environment but unable to sporulate failed to survive or leave progeny. This scenario reinforces the argument that kinorhetic transformation has been selected evolutionarily and processes that propel organisms outside of their h0meostatic situations have been essential for survival from the earliest beginnings.

### 5.4 Human reproduction

**FIGURE 7:**
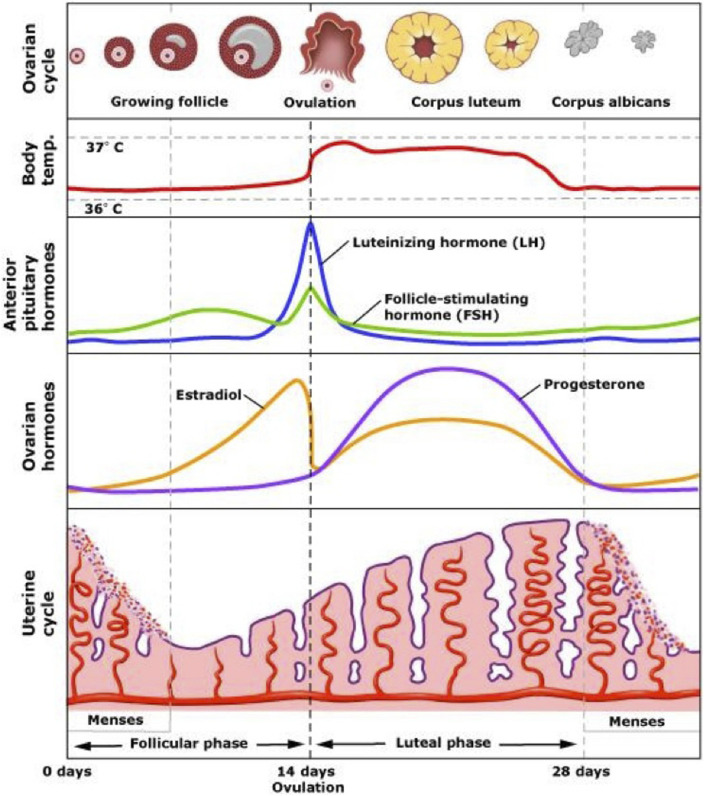
The human ovarian (menstrual) cycle. The morphology of the ovarian follicles (top row) is a series of states that closely adheres to the general scheme for sequential control shown in Figure 6. The follicle and egg grow until reaching a state of maturity after which the egg is ovulated. After ovulation the follicle transforms into a new structure, the corpus luteum, characterized by fatty cells that secrete large amounts of progesterone. The corpus luteum decays to the inactive corpus albicans. The third and fourth lines illustrate hormonal changes over the cycle. Estrogen rises along with the growth of the follicles, reaching a level that provides a positive feedback for secretion of LH from the pituitary (and GnRH from the hypothalamus). The spiking of LH is the proximate cause of ovulation.

Not surprisingly the vast literature of human physiological studies provides a wealth of information about homeostasis and kinorhesis. Unlike many other species, humans have the ability to control many aspects of their environments, so we do not utilize some exquisite kinorhetic adaptions that occur in other animals. For example, human migrations lack the physiological drama of salmon migrations. However, human reproduction provides many examples of kinorhetic transformations that propel the creation of a new generation. These operate in parallel with the homeostasis of the parents, and eventually the homeostasis of the offspring. One illustrative kinorhetic process is the growth and ovulation of the eggs (Figure 7).

The ovulatory cycle (aka menstrual cycle) is governed by continuous interactions among hormones that are secreted from the brain, pituitary gland, and ovaries (Marshall and Reame, 2015). The hypothalamus of the brain secretes a small neuropeptide, gonadotropin-releasing hormone (GnRH) that stimulates the pituitary gland. The pituitary gland secretes 2 gonad stimulating hormones, follicle stimulating hormone (FSH) and luteinizing hormone (LH). FSH supports the growth of the ovarian follicles, which contain the developing eggs. The follicular cells surrounding the eggs provide nutritive support and secrete estrogen.

As the egg and surrounding cells grow under the influence of FSH they secrete increasing amounts of estrogen, which keeps LH levels low by negative feedback. When the follicle reaches maturity estrogen levels reach a high level and the system switches into a positive feedback mode where estrogen causes the hypothalamus to secrete waves of GnRH, which drives a series of spikes of LH. These LH spikes cause explosive growth and development of the follicle, which expels the egg in the process of ovulation. After expelling the egg the follicular cells grow and transform into a highly active steroid hormone factory (corpus luteum) secreting large amounts of progesterone. These hormones cause the uterus to grow and develop a rich blood supply so that the embryo can implant itself if fertilization occurs. Implantation leads to placentation, organogenesis, and fetal growth in a series of kinorhetic processes that organize viviparous mammalian reproduction.

**FIGURE 8:**
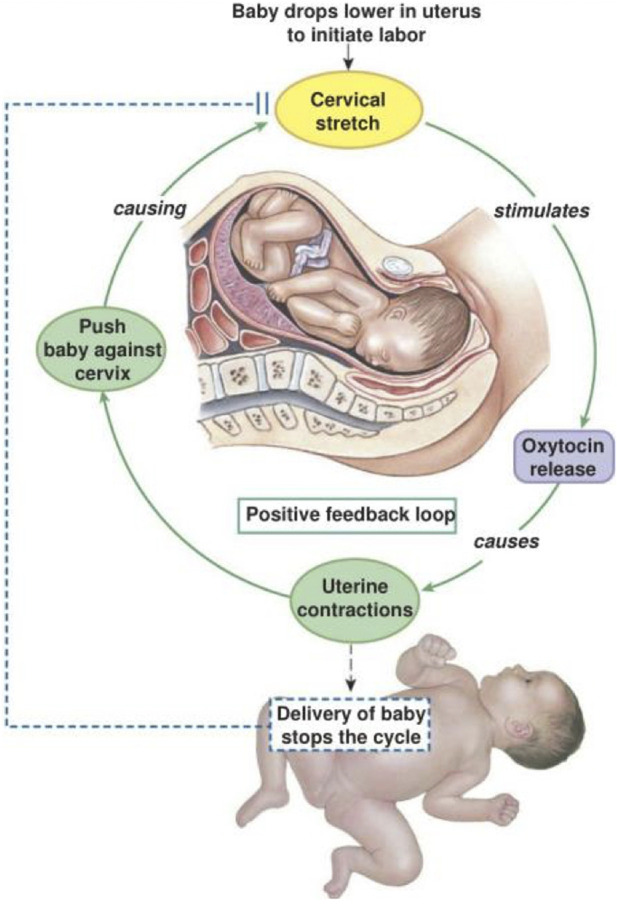
Childbirth illustration of positive feedback. After labor has initiated oxytocin is increasingly secreted by the posterior pituitary. Oxytocin stimulates both the force and frequency of uterine contractions. Pressure receptors in the uterine cervix are stimulated and signal the posterior pituitary to increase oxytocin secretion. As this process repeats and becomes more exaggerated the fetus is forced against, and ultimately through the cervix. Delivery of the fetus and placenta break the cycle of increasing oxytocin once birth is completed.

Like the ovulatory process, childbirth exemplifies the transformative consequences of kinorhetic regulation in the context of human reproduction. Late in pregnancy changes in the absolute and relative levels of estrogens, progesterone, and relaxin begin the sequence of events that culminate in labor and delivery (Gibb et al., 2006). Progesterone and relaxin decline while estrogens increase. The placenta begins to secrete increasing amounts of prostaglandins. High relative estrogen and prostaglandin initiates changes in the uterus and cervix that prepare the womb for birth. In combination with changes in hormones that are secreted by the placenta such as estrogens and prostaglandins the hypothalamus begins to secrete oxytocin as labor begins. Oxytocin does not initiate natural labor, but once the first phase of labor has begun a positive feedback loop between the uterus and the hypothalamus increases the amount and frequency of oxytocin surges (Figure 8). These oxytocin surges strengthen and lengthen uterine contractions, which increases pressure on the cervix and uterus, leading to greater stimulation of oxytocin secretion. Ultimately, delivery of the fetus and placenta bring a limit to the escalation of oxytocin feedback.

The physiology of pregnancy illustrates the extreme demands that kinorhetic processes can put on homeostasis (Thornburg et al., 2006). Maternal adaptations of homeostasis during pregnancy encompass all or nearly all organ systems. For example, the immune system adapts to the presence of an embryo and fetus that express foreign antigens (Abu-Raya et al., 2020) and the digestive and metabolic systems adapt to increased energy demand, including a basal metabolic rate that is elevated by as much as 50%. Many systems, including respiratory, cardiovascular, and renal continue to adjust homeostatically throughout pregnancy concurrent with increasing demands caused by growth of the fetus and placenta.

## 6 Conclusion and perspectives

Homeostasis is a powerful and essential governing principle not only for classical mammalian physiology, but also for the entire scope of functional biology across the Kingdoms. As a theory homeostasis derives its power from its particularity. That is, homeostasis means “stability of the internal milieu”. That particularity guides physiologists to answer specific questions, such as “what is internal vs. external in this context?”, “how stable must a particular variable be to remain within homeostasis?”, “how are variations sensed?”, “where do the controlling signals originate?”, and many others? The meaning of homeostasis has grown over time as new discoveries have been made. This growth means that homeostasis now applies to all species, not just humans and other mammals that concerned Cannon (1939). Related names for homeostasis have been offered, which also have opened up its meaning to encompass the scope of changes that are compatible with stability of the organism.

But growing the meaning of homeostasis (and related names) cannot mean applying the concept to physiological processes that contradict its inherent meaning. Because homeostasis has a particular meaning it would be absurd to say something along the lines of “metamorphosis is homeostatic”. Aspects of homeostasis certainly endure during metamorphosis, but the point of metamorphosis is transformation, not stability. Different language has been needed to encompass the non-homeostatic physiology of transformations such as metamorphosis, sexual maturation, parturition, and others that happen during the course of any given life history. Kinorhesis is a term that expresses that physiological processes propel (kino-) the flow (rheo) of organismal transformations. Consequently it is perfectly reasonable to say that “metamorphosis is kinorhetic.”

As I have pointed out, homeostasis and kinorhesis coexist. Natural selection has mixed and matched homeostatic and kinorhetic processes in ways that range from the sublime (butterfly emergence) to the ridiculous (praying mantis chewing the head off her mate). Evolution is not dogmatic. Natural selection employs the mechanisms underlying homeostasis and kinorhesis according to their utility for surviving and reproducing.

Kinorhesis is heuristically useful because it employs known physiological mechanisms to explain biological events that otherwise invite teleological and vitalistic explanations (Allen and Neal, 2020; Lennox and Kampourakis, 2013; Turner, 2013). The impulse to talk about biological “purposes”, “goals”, “wants”, “desires” persists even after many decades of science based in physical materialism (Turner, 2013; Turner, 2017; Billman, 2020). For example, it is not unusual to hear it said that animals migrate for the “purpose” of finding food or water, or calling the drive to reproduce a “life force”. Kinorhesis replaces teleologic and vitalistic purposes and goals with known physiological processes of positive feedbacks and sequential controls that are available to natural selection and scientific experimentation.

Teleology is very common and has recently been called “ineliminable” in biology (Allen and Neal, 2020). A more correct view is that teleological reasoning, *per se*, is indefensible, but the habit of using teleological language is irresistible (you almost certainly would find some in this paper). Kinorhesis combines non-teleological causal mechanisms and vocabulary that explain transformative life events that can appear to be purposive or goal-oriented. The very common use of teleological language by scientists is usually a form of linguistic “code-switching”. Teleological language makes for easy and concise communication, but we automatically switch modes out of teleology in order to do experiments and construct conceptual models of the processes we discover. Evidence of this tendency to code-switch is that even when teleological phrasing creeps into biological papers, one does not find diagrams of physiological mechanisms that include “purposes” or “goals” as causes to explain biological events.

Kinorhetic mechanisms accomplish remarkable physiological transformations using well known processes of positive feedbacks and sequential control that are implemented by employing discoverable macromolecular and metabolic substrates. Therefore, kinorhesis is a consistently heuristic way to approach physiological problems, just as is homeostasis.

Teaching of physiology at even the most introductory levels has made good use of the concept of homeostasis. In particular, students learn early in biology classes the notion that negative feedback provides physiological stability. But there is a huge vacancy left because there are obviously many things that happen in physiology that are not explained by homeostasis. Puberty is a familiar physiological process that intuitively does not fit a definition of stability. Incorporating kinorhesis alongside homeostasis can establish a basis for teaching about physiological transformations by reference to kinorhetic mechanisms (positive feedback and sequential control) that are the equivalent of homeostatic mechanisms. Having learned these fundamentals students will be able to conceptualize specific physiological adaptations and life history events with a mechanistic foundation in mind. Understanding biological phenomena through theories such as homeostasis and kinorhesis is important for professional scientists and for students of biology or medicine.

## Data Availability

The original contributions presented in the study are included in the article/supplementary material, further inquiries can be directed to the corresponding author.
